# On Medical Electricity

**Published:** 1811-08

**Authors:** 


### *To the Editors of the Medical and Physical Journal.*

GENTLEMEN,

I BELIEVE such subjects as are in any way connected with Medicine will find admission into your periodical undertaking. As *electricity* has been found lately to have alleviated some very distressing diseases, and to have removed others of a less formidable nature; I am induced to recommend it to your consideration. That its effects are very powerful, is beyond doubt; and when they are directed by the advice of medical men, but more particularly when under der their immediate management, we have reason to expect the most favourable results. Electricity is a subject, hitherto, but very imperfectly understood; at the same time, I cannot hesitate to say, that the treatises of the present day are mere recapitulations of the past. It will be well then to revive the subject in all its parts, and submit it to a thorough investigation, which in the present improved state of science may be more easily accomplished. Many a valuable remedy has been ultimately laid aside, because more has been expected from it than it could possibly accomplish; and because it could not cure every disorder to which it had been most injudiciously applied.

If gentlemen, whether of the medical profession or not, would favour you with the results of their investigations and experiments; Electricity, long neglected by the greatest number of practitioners, would become, with its surprizing phenomena, fixed on a more certain basis.

As it is necessary to have the best machine, I would propose the following question.

What is the most advantageous form for an electrical machine, and what are the reasons for such a peculiarity of construction? R.

